# AI-Based Predictive Models for Cardiogenic Shock in STEMI: Real-World Data for Early Risk Assessment and Prognostic Insights

**DOI:** 10.3390/jcm14113698

**Published:** 2025-05-25

**Authors:** Elena Stamate, Anisia-Luiza Culea-Florescu, Mihaela Miron, Alin-Ionut Piraianu, Adrian George Dumitrascu, Iuliu Fulga, Ana Fulga, Octavian Stefan Patrascanu, Doriana Iancu, Octavian Catalin Ciobotaru, Oana Roxana Ciobotaru

**Affiliations:** 1 Department of Morphological and Functional Sciences, Faculty of Medicine and Pharmacy, “Dunarea de Jos” University of Galati, 35, Al. I. Cuza Street, 800216 Galati, Romania; elena.stamate@ugal.ro; 2 Department of Electronics and Telecommunications, “Dunarea de Jos” University of Galați, 800008 Galati, Romania; 3 Department of Computer Science and Information Technology, “Dunarea de Jos” University of Galați, 800008 Galati, Romania; mihaela.miron@ugal.ro; 4 Division of Hospital Internal Medicine, Department of Medicine, Mayo Clinic Florida, 4500 San Pablo Rd S, Jacksonville, FL 32224, USA; 5 Department of Medical, Faculty of Medicine and Pharmacy, “Dunarea de Jos” University of Galati, 35, Al. I. Cuza Street, 800216 Galati, Romania; iuliu.fulga@ugal.ro; 6 Department of Clinical Surgical, Faculty of Medicine and Pharmacy, “Dunarea de Jos” University of Galati, 35, Al. I. Cuza Street, 800216 Galati, Romania; ana.fulga@ugal.ro (A.F.); octavian.ciobotaru@ugal.ro (O.C.C.); 7 Faculty of Medicine and Pharmacy, Dunarea de Jos University of Galati, 35 AL Cuza St, 800010 Galati, Romania; octav974@gmail.com (O.S.P.);; 8 Department of Clinical Medical, Faculty of Medicine and Pharmacy, “Dunarea de Jos” University of Galati, 35, Al. I. Cuza Street, 800216 Galati, Romania; roxana.ciobotaru@ugal.ro

**Keywords:** STEMI, cardiogenic shock, machine learning, early triage, angiography prioritization, predictive models

## Abstract

**Background:** Cardiogenic shock (CS) is a life-threatening complication of ST-elevation myocardial infarction (STEMI) and remains the leading cause of in-hospital mortality, with rates ranging from 5 to 10% despite advances in reperfusion strategies. Early identification and timely intervention are critical for improving outcomes. This study investigates the utility of machine learning (ML) models for predicting the risk of CS during the early phases of care—prehospital, emergency department (ED), and cardiology-on-call—with a focus on accurate triage and prioritization for urgent angiography. **Results:** In the prehospital phase, the Extra Trees classifier demonstrated the highest overall performance. It achieved an accuracy (ACC) of 0.9062, precision of 0.9078, recall of 0.9062, F1-score of 0.9061, and Matthews correlation coefficient (MCC) of 0.8140, indicating both high predictive power and strong generalization. In the ED phase, the support vector machine model outperformed others with an ACC of 78.12%. During the cardiology-on-call phase, Random Forest showed the best performance with an ACC of 81.25% and consistent values across other metrics. Quadratic discriminant analysis showed consistent and generalizable performance across all early care stages. Key predictive features included the Killip class, ECG rhythm, creatinine, potassium, and markers of renal dysfunction—parameters readily available in routine emergency settings. The greatest clinical utility was observed in prehospital and ED phases, where ML models could support the early identification of critically ill patients and could prioritize coronary catheterization, especially important for centers with limited capacity for angiography. **Conclusions:** Machine learning-based predictive models offer a valuable tool for early risk stratification in STEMI patients at risk for cardiogenic shock. These findings support the implementation of ML-driven tools in early STEMI care pathways, potentially improving survival through faster and more accurate decision-making, especially in time-sensitive clinical environments.

## 1. Introduction

Cardiogenic shock (CS), characterized by insufficient cardiac output leading to organ dysfunction, remains a significant challenge for cardiovascular specialists. The definition of CS remains inconsistent across guidelines, medical organizations, and clinical research. To address this, experts have proposed two standardized definitions, namely a clinical definition, which describes CS as a cardiac condition causing sustained tissue hypoperfusion, both clinically and biochemically, regardless of blood pressure, and a research definition, which defines CS as cardiac dysfunction leading to a systolic blood pressure below 90 mmHg for at least 30 min or requiring vasoactive medications, inotropic agents, or mechanical support to maintain blood pressure, together with evidence of tissue hypoperfusion [1,2]. CS manifests through a wide range of clinical presentations representing a complex hemodynamic syndrome [3]. CS complicating ST-segment elevation myocardial infarction (STEMI-CS) is associated with a high mortality rate of 35–50% within the first 30 days [4,5,6]. Moreover, the impairment of quality of life and functional capacity in patients following STEMI-CS places a significant burden on healthcare system resources [7,8,9].

**Figure 1:**
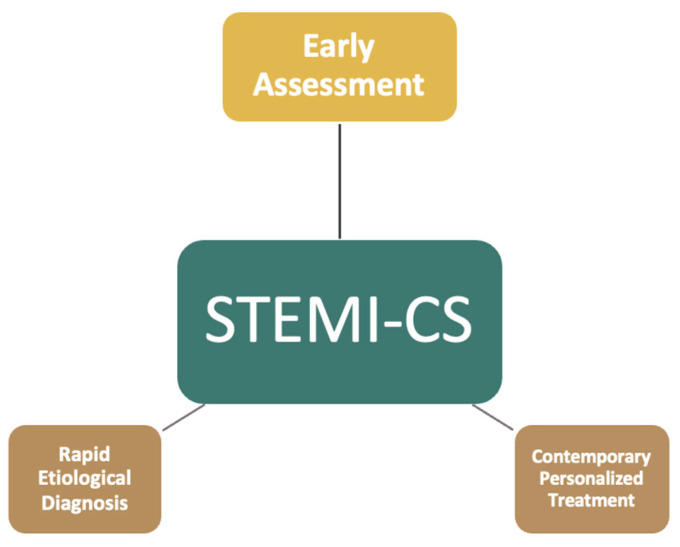
Stepwise approach to risk assessment in STEMI-CS patients. CS complicating ST-segment elevation myocardial infarction: STEMI-CS.

Recently, studies in this field have focused on the early identification and better understanding of STEMI-CS through the implementation of well-structured protocols, teamwork, contemporary treatment methods, rapid etiological diagnosis, and early assessment of hemodynamic status [7,10,11,12], as illustrated in Figure 1. If the stages of CS, according to the new Society for Cardiovascular Angiography and Interventions (SCAI) SHOCK classification, represent a dynamic process, then risk assessment for progression to CS should also follow a dynamic stepwise approach [13,14]. This creates a system in which the patient is regularly reassessed to identify the exact moment of CS onset, which is often underdiagnosed. However, timely interventions by dedicated shock teams can help minimize subsequent damage and optimize patients’ chances for a successful outcome [1,15].

**Figure 2:**
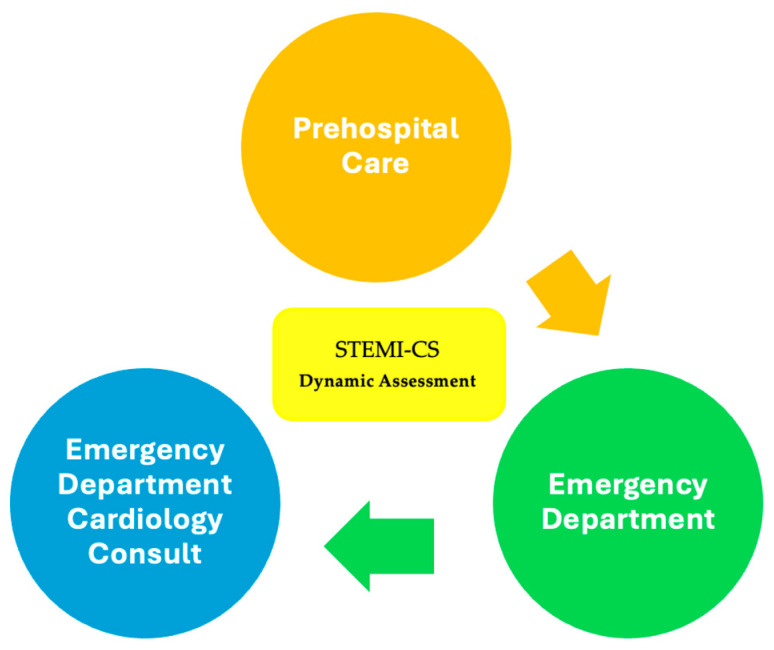
Progressive early risk evaluation in STEMI-CS: A structured approach across care phases.

This study focuses on the early identification and evaluation of parameters associated with STEMI-CS risk, employing a stepwise approach that spans the prehospital phase through to the ED cardiology consultation, as illustrated in Figure 2. In high-volume centers with limited catheterization lab availability, rapid risk stratification can help prioritize angiographic intervention, potentially reducing mortality through timely, targeted decisions. While previous studies have examined isolated clinical, biological, or imaging variables, none have integrated these factors into a unified, phase-specific framework that reflects the evolving risk dynamics throughout the early care continuum. To address this gap, we propose a comprehensive model based on machine learning that dynamically assesses risk across care stages, supporting the earlier recognition of CS and more personalized therapeutic strategies.

The use of artificial intelligence (AI) in predictive modeling across the early stages of STEMI care offers the potential to improve outcomes in STEMI-CS patients by enabling faster, personalized implementation of optimal therapy, regardless of the timing of assessment within the care continuum [16,17,18]. Leveraging its capacity to process complex, multi-parametric data, AI provides predictive accuracy that often exceeds traditional statistical approaches [19,20,21,22], thus supporting the timely identification of patients requiring urgent hemodynamic support [23,24]. By enabling earlier and individualized therapeutic decisions, this strategy may help reduce STEMI-CS-related morbidity and mortality [25] and improve patients’ functional recovery while also contributing to a more efficient allocation of healthcare resources [26,27,28,29]. By tailoring treatment to individual patient profiles, this approach also has the potential to reduce the economic burden associated with prolonged hospital stays, complications, and costly interventions, such as mechanical circulatory support, while significantly enhancing the quality of life for STEMI-CS patients [1,7,25,26,30,31].

## 2. Materials and Methods

### 2.1. Dataset

**Figure 3:**
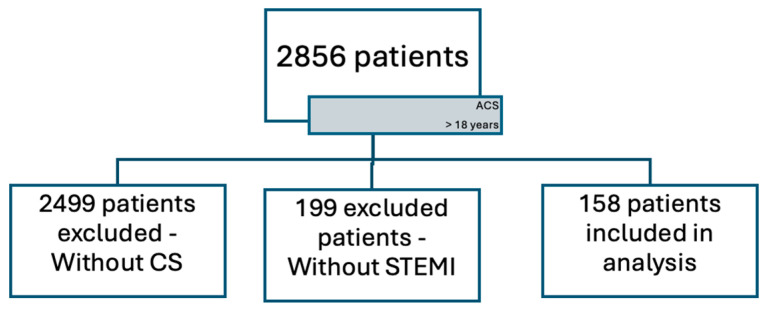
Flowchart of patient selection. Cardiogenic shock: CS.

We retrospectively analyzed data from 2856 adult patients (>18 years) hospitalized with a diagnosis of acute coronary syndrome (ACS), according to the established criteria [32]. All patients received care between 2019 and 2022 in the Cardiology Unit of the University Emergency Hospital of Bucharest, Romania. Of these, 357 patients developed cardiogenic shock (CS), as defined by current clinical standards [3]. Further exclusion of cases unrelated to ST-elevation myocardial infarction (STEMI) yielded a final cohort of 158 patients with confirmed STEMI-CS, as shown in Figure 3, which illustrates the patient selection flowchart from initial ACS admissions to the final STEMI-CS cohort. Patients were further divided into two groups, namely patients with CS present at admission and patients with CS post-admission. Patients without CS were excluded, as the goal was to develop a model for identifying risk factors and early predictors within the STEMI-CS population only.

This was a retrospective, observational, single-center study. Data were anonymized to preserve confidentiality. Ethical principles were followed in accordance with the Declaration of Helsinki. Informed consent was not required due to the retrospective, anonymized, and non-interventional nature of the study.

Inclusion Criteria:

1. Acute coronary syndrome complicated by cardiogenic shock in patients who received care in the Cardiology Unit of the University Emergency Hospital of Bucharest.
2. Age > 18 years.

Exclusion Criteria:

1. Patients with cardiogenic shock due to etiologies other than STEMI, such as unstable angina [32], NSTEMI, myocarditis [33], pulmonary embolism, infectious endocarditis [34], and non-ischemic cardiomyopathy.
2. Medical records with missing hospitalization data.
3. Patients who requested to be discharged against medical advice.
4. Patients with end-stage liver disease.
5. Patients with a diagnosis of sepsis [35].
6. Patients with other severe infections without a diagnosis of sepsis.
7. Patients with severe malnutrition.
8. Patients receiving large-volume blood transfusions.
9. Patients with a diagnosis of active malignancy.
10. Patients with coagulation disorders, such as patients with a diagnosis of thrombophilia and patients with coagulopathy.

Patients with NSTEMI and unstable angina were excluded in order to reduce population heterogeneity and allow for the development of focused, phase-specific predictive models in STEMI, where management is time-sensitive and standardized (i.e., immediate reperfusion).

### 2.2. Analyzed Variables

We examined patients’ records and mapped the clinical data to three key care phases relevant for early risk assessment, namely the prehospital phase, emergency department (ED) phase, and cardiology consultation phase in the ED.

**Figure 4:**
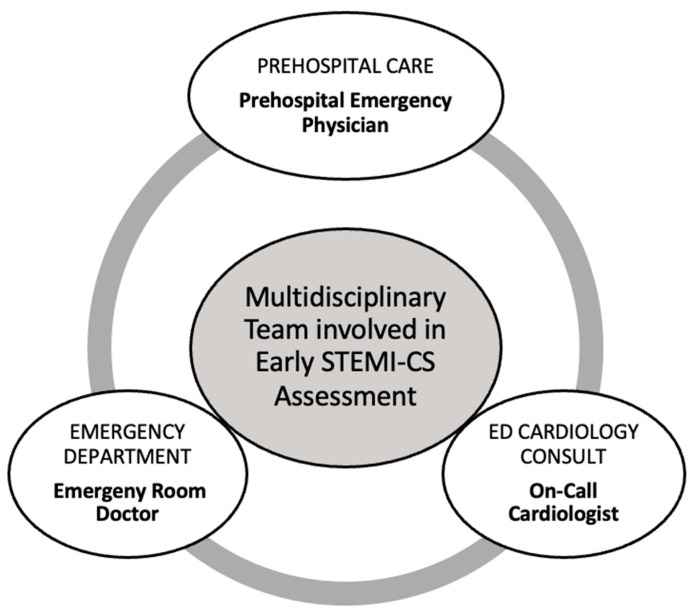
Multidisciplinary team involved in early STEMI-CS patient’s assessment across different phases of care.

Following this structured classification, we described the multidisciplinary team involved in STEMI-CS patients’ management, detailing their roles with each stage, as illustrated in Figure 4 below. This collaborative model facilitates prompt decision-making and efficient triage.

The variables analyzed in these early stages included the following:

o Demographic and Clinical Data: Age, sex, time from symptom onset, heart rate, and Killip class (according to the definition [36];
o Cardiovascular Risk Factors: Diabetes (according to the definition [37]), hypertension (as defined by 2024 ESC Guidelines [38]), dyslipidemia: new diagnosis and history of dyslipidemia under treatment (based on the 2019 ESC definition [39]), and active smoking at the time of admission;
o ECG Features: ECG rhythm (sinus rhythm, atrial fibrillation, ventricular tachycardia, ventricular fibrillation, and junctional rhythm), conduction abnormalities, localization of ST elevation, ST elevation in aVR, QRS duration, Q wave presence, and reciprocal ST depression;
o Laboratory Findings (in ED): Hemoglobin, leukocyte count, troponin, creatine kinase–MB isoenzyme (CKMB), creatine kinase isoenzyme (CKI), glucose, creatinine, potassium (K), sodium (Na), aspartate aminotransferase (AST), ALT (alanine aminotransferase), urea nitrogen (BUN), and fibrinogen;
o Echocardiographic Data (ED cardiology consultation phase): left ventricular ejection fraction (LVEF) (EF > 50, EF 40–50, EF < 40), mitral regurgitation, right ventricular (RV) dysfunction, left ventricular (LV) thrombosis, LV aneurysm, pericardial effusion, and mechanical complications.

The selected variables were chosen based on their routine availability at the initial point of care and their known clinical relevance for the early prediction of cardiogenic shock prior to revascularization. In developing the predictive models, we prioritized simple, routinely collected clinical, biochemical, electrocardiographic, and echocardiographic parameters, reflecting current standards in cardiovascular risk assessment. The Killip classification at admission served as a key clinical marker, given its established prognostic value and its ability to support rapid decision-making regarding urgent coronary intervention or hemodynamic stabilization. Biochemical markers such as creatinine, CKI, potassium, and hemoglobin were included to reflect systemic metabolic status and the degree of cardiac and renal impairment. ECG abnormalities and echocardiographic signs of ventricular dysfunction were similarly incorporated, given their capacity to signal early cardiac compromise.

Variables with a high proportion of missing data—such as NT-proBNP, lactate, and oxygen saturation—were excluded from the final analysis to maintain model integrity.

The selection of variables for model development was based on both clinical relevance and practical applicability during the early phases of STEMI care. To enable accurate and interpretable risk stratification, we aimed to include parameters that reflect multiple dimensions of the patient’s condition, such as the following:

- Hemodynamic status (e.g., Killip class, heart rate) [36,40];
- Metabolic and renal function (e.g., creatinine, potassium) [41];
- Electrical instability (e.g., ECG rhythm, ST changes, aVR elevation) [42,43,44];
- Functional cardiac impairment (e.g., LVEF, RV dysfunction, mitral regurgitation) [41,45].

This multidimensional approach was chosen to ensure that the predictive model could capture the complex pathophysiology of cardiogenic shock while remaining feasible for real-time use. All variables were selected based on routine availability at the initial point of care, without the need for specialized testing or imaging. This approach aligns with current ESC guidelines for STEMI management [43] and reflects clinical priorities for the early triage and treatment of high-risk patients, as outlined in established risk models such as GRACE [40].

Variables with a high rate of missing data—such as NT-proBNP, lactate, and oxygen saturation—were excluded from the final analysis to avoid bias and preserve model integrity. However, their inclusion is planned in future prospective validation studies, where standardized data collection will allow for more comprehensive modeling.

### 2.3. Data Preprocessing

For each subsequent phase, the variables available in the earlier phases were also included in the analysis and AI modeling, with the ED cardiology phase having the most comprehensive dataset for analysis.

### 2.4. Statistical Analysis

The statistical analysis was conducted on data collected from patients across three distinct critical phases encompassing patients’ evaluation, namely the prehospital phase, emergency department phase, and cardiology consultation phase in the emergency department. In the first phase of our study, we employed Random Forest for feature selection due to its numerous advantages in handling complex medical datasets. RF is a machine learning technique that constructs multiple decision trees and aggregates their predictions, making it a robust and reliable tool for extracting the most relevant characteristics for CS prediction. One of the primary reasons for using RF is its ability to capture complex, non-linear relationships between variables without requiring prior assumptions about their interactions. Unlike traditional statistical methods, RF can efficiently identify the most relevant clinical, hemodynamic, and angiographic parameters associated with cardiogenic shock, even when these features exhibit intricate interdependencies. Another key advantage of RF is its automated feature selection process. By computing out-of-bag (OOB) error rates and measuring feature importance, RF ranks the most significant parameter predictors, allowing us to select the top seven most relevant parameters. From a statistical perspective, the inclusion of non-informative or redundant features can lead to increased model complexity, higher estimation variance, and diminished generalization capability. This issue is commonly observed in machine learning models, where the performance tends to deteriorate when the number of predictors exceeds the optimal number leading to overfitting. By focusing only on the most relevant parameters, feature selection helps simplify the model, improves accuracy, and makes the results easier to interpret and more stable.

To evaluate the predictive strength of the model, we used the accuracy metric, which measures the proportion of correct predictions. This metric reflects the overall predictive ability of the model.

To account for both precision and sensitivity, we calculated the F1-score. This metric is especially important in medical applications, as it balances the correct identification of cardiogenic shock cases (sensitivity) and the accuracy of positive predictions (precision).

This ensures that both false positives and false negatives are appropriately minimized.

Furthermore, we evaluated the model’s robustness by calculating the 95% confidence intervals (CIs) for accuracy, ensuring the model’s precision in clinical settings. This is very important for determining the model’s reliability in a clinical context, where small margins can have significant implications for patient care.

Following the feature selection process, we incorporated logistic regression to evaluate the predictive significance of the selected parameters and to provide clear interpretations of their contributions to the model. However, LR assumes linear relationships between predictors and the outcome, which may limit its ability to model complex dependencies. RF, in contrast, captures complex feature interactions that LR may miss, allowing for better performance in non-linear datasets.

To determine how well the selected parameters explain the variability in cardiogenic shock outcomes, multicollinearity was evaluated using the variance inflation factor (VIF), ensuring that the selected features are not overly correlated with each other, which could distort the regression analysis. Low VIF values, typically below 10, suggest that the selected features are independent enough to maintain model stability and interpretability. This step is especially critical in clinical settings, where the understanding of individual predictors can inform decision-making and treatment strategies.

After training both the Random Forest and logistic regression models on the selected features, McNemar’s test was applied to compare the models’ performance and determine whether the differences in their binary predictions were statistically significant. The test helped evaluate if including interaction terms in logistic regression or comparing it with Random Forest led to meaningful changes in the classification of cardiogenic shock cases. While McNemar’s test does not assess overall accuracy, it highlights whether the models make significantly different decisions in the same cases, providing valuable insight into the models’ comparative strengths.

In addition to evaluating model discrimination and agreement, calibration was examined to determine how closely the predicted probabilities reflected actual outcomes. While metrics such as the AUC and McNemar’s test provide valuable information about the model’s ability to distinguish between outcomes and detect differences in classification, they do not measure the accuracy of predicted risk levels. To address this, Brier scores were calculated. The Brier score quantifies the mean squared difference between predicted probabilities and actual outcomes, with lower scores indicating better calibration. This step is essential in clinical applications, where accurate and reliable risk estimates support better informed decision-making and improve patient care.

### 2.5. Machine Learning Models

In this study, we selected eleven ML algorithms, including tree-based (Extra Trees—ET; Random Forest—RF; decision tree—DT), probabilistic (quadratic discriminant analysis—QDA; naïve Bayes—NB), margin-based (support vector machine—SVM), linear models (logistic regression—LR; Ridge Classifier—RC), distance-based (K-nearest neighbors—KNN), and boosting methods (gradient boosting—GBC, AdaBoost—ADA), to predict the early-stage risk of cardiogenic shock, represented as a binary outcome (1 = at risk; 0 = not at risk. These ML models were chosen for their diverse strengths in capturing complex patterns in medical data. Tree-based models (ET, RF, DT) are robust in overfitting and handling non-linear relationships effectively. Probabilistic models (QDA, NB) provide valuable probabilistic outputs, essential for medical risk assessment. An SVM was selected for its ability to separate high-risk and low-risk cases in high-dimensional spaces. Linear models (LR, RC) are interpretable and work well with binary classification tasks, while KNN excels in cases with complex, local patterns. Finally, boosting methods (GBC, ADA) were chosen for their ability to improve performance by combining weak learners, particularly in imbalanced datasets.

The ML-based framework was implemented in Google Collab (Alphabet Inc., Mountain View, CA, USA) using Python (The Python Software Foundation, Beaverton, OR, USA) version 3.11.11. Key libraries used in this analysis included pandas, matplotlib, seaborn, and scikit-learn. The clinical datasets were split into training (80%) and testing (20%) sets using train_test_split from sklearn.model_selection.

To ensure robust model selection and reduce overfitting, a 5-fold cross-validation was applied exclusively to the training data. Missing data were handled through listwise deletion to maintain consistency and prevent data leakage across folds. Hyperparameters were optimized based on average performance across validation folds. After tuning, the final models were retrained on the entire training set and evaluated on the hold-out test set.

The ML models were configured with specific hyperparameters to ensure consistency across evaluations. LR was set with max_iter = 1000 to ensure convergence. Ensemble models such as RF, ET, GBC, and ADA, were configured with n_estimators = 100 and random_state = 42. The SVM used the RBF kernel with the regularization parameters of C = 1.0. KNN was set with n_neighbors = 5. RF was used with alpha = 1.0 and solver = ‘auto’, QDA was configured with reg_param = 0.0, store_covariance = False, and tol = 1.0 × 10^−4^. NB was used with var_smoothing = 1 × 10^−9^. All models were trained on data standardized using StandardScaler.

For evaluation, each model was trained on the training dataset and then used to make predictions on the test dataset. Performance metrics including accuracy, precision, recall, F1-score, and MCC were calculated. A confusion matrix was also generated to analyze prediction outcomes.

### 2.6. Framework

**Figure 5:**
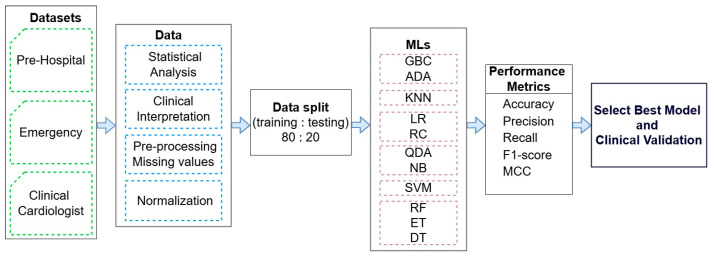
The machine learning-based framework for cardiogenic shock prediction. GBC: Gradient Boosting Classifier; ADA: AdaBoost Classifier; KNN: K-Nearest Neighbors; LR: Logistic Regression; RC: Ridge Classifier; QDA: Quadratic Discriminant Analysis; NB: Naive Bayes; SVM: Support Vector Machine; RF: Random Forest; ET: Extra Trees Classifier; DT: Decision Tree.

This study represents an interdisciplinary collaboration between engineers and clinicians, integrating medical knowledge with AI techniques aiming to improve the assessment and early prediction of cardiogenic shock. The framework for cardiogenic shock detection based on machine learning is illustrated in Figure 5, highlighting the key steps of our research, which are as follows:

- Three datasets (marked with a green color) from the prehospital phase, emergency department phase, and ED cardiology consultation phase with real clinical data were collected by providers from the Cardiology Department of the University Emergency Hospital of Bucharest, Romania, in accordance with the inclusion and exclusion criteria outlined in the Section 2.1.
- Advanced statistical analysis and clinical interpretation were performed to select the most relevant clinical parameters for predicting cardiogenic shock, reducing dimensionality while preserving predictive performance. Multicollinearity was evaluated using the variance inflation factor, and McNemar’s test assessed interaction effects and model consistency. Model performance was measured using accuracy for overall correctness and the F1-score to balance precision and sensitivity, minimizing both false positives and false negatives. Then, 95% confidence intervals (CIs) for accuracy were computed to assess robustness in clinical settings. Following parameter selection, logistic regression was used to evaluate the explanatory power of individual predictors, while Random Forest captured non-linear interactions to enhance performance in complex datasets.
- Data preprocessing steps were applied, including the handling of missing values and feature normalization to ensure data quality and consistency.
- The pre-processed data were split into training and testing sets in an 80:20 ratio for the training and testing of eleven ML models (marked with a purple color). These models were evaluated using standard performance metrics (accuracy—ACC; precision; recall; F1-score; and Matthews correlation coefficient—MCC) to identify the most accurate and clinically relevant model.
- Clinical validation was conducted for the best-performing ML model to assess its applicability and reliability in real-world medical settings.

## 3. Results

### 3.1. Study Population and Cardiovascular Risk Factors

A total of 158 patients with STEMI—CS fitting the inclusion and exclusion criteria and evaluated between 2019 and 2022 were included in the database. We found a significant difference in sex and age between STEMI patients with CS present on admission and patients with CS developed post-admission. The average age of patients was 74 years in the STEMI group with CS present at admission and 65 years in the STEMI patients with CS post-admission. Among patients that were admitted with a diagnosis of CS, males and females were equally represented (48.43% vs. 50.57%), but more males than females (76% vs. 26%; OR: 0.97 vs. 0.31) were present within the cohort that developed CS after admission, as illustrated in Table 1.

When comparing cardiovascular risk profiles, several differences can be observed between STEMI patients with CS present at admission and CS post-admission. Notably, patients who developed CS later during hospitalization exhibited a higher prevalence of traditional cardiovascular risk factors. Diabetes mellitus was present in 27 (38.02%) patients in the progression group compared to 23 (26.43%) patients in those who were admitted with CS. Similarly, hypertension was significantly more prevalent in the progression group, with 55 patients vs. 31 patients (77.46% vs. 36.78%), as was smoking, with 30 patients vs. 27 patients (49.29% vs. 31.03%). In contrast, dyslipidemia showed a high prevalence in both groups, with a slightly higher proportion among those who received care directly with CS, at 65 patients vs. 50 patients (74.71% vs. 71.83%). These findings suggest that patients who progress to CS during hospitalization tend to have a more heavily burdened cardiovascular risk profile, potentially contributing to a more gradual and complex decompensation process, as shown in Table 1.

### 3.2. Model Performance

Based on the general theoretical framework, each phase provides distinct statistical evidence regarding key parameters influencing cardiogenic shock risk, underscoring their importance in predicting patient outcomes throughout clinical care.

#### 3.2.1. Predictive Parameters for Prehospital Phase

This model is based on 17 key parameters that are readily available during patient evaluation in the field. The model incorporates a combination of demographic factors, pre-existing conditions, clinical presentation, and electrocardiographic data.

The key variables identified through feature selection using Random Forest analysis, proven significant in predicting cardiogenic shock risk, are Killip at presentation, age, EKG rhythm at presentation, pain onset, sex, HR at presentation, and ST-segment elevation in aVR, as they provide essential predictive information for risk assessment and clinical decision-making. To predict the risk of cardiogenic shock, we employed Random Forest as the primary predictive model.

However, to evaluate the explanatory power of the selected features and evaluate the individual contribution of each parameter, we incorporated logistic regression. This dual approach enables both predictive accuracy (through RF) and interpretability (through LR).

The predictive performance of the models was evaluated using several metrics, including accuracy, sensitivity, specificity, F1-score, area under the curve (AUC), and McNemar’s test. The comparison between Random Forest and logistic regression is shown in Table 2 below.

The results indicate that Random Forest and logistic regression perform similarly in the prehospital phase. Random Forest achieves slightly higher accuracy (77.42% vs. 74.19%) and sensitivity (77.78% vs. 72.22%), suggesting a marginally better ability to correctly identify high-risk patients. However, logistic regression exhibits a higher AUC (0.8248 vs. 0.7735), indicating better overall discriminatory power in distinguishing patients at risk of cardiogenic shock. In terms of calibration, logistic regression also outperforms Random Forest, with a lower Brier score of 0.1738 compared to 0.1927 for Random Forest, reflecting more accurate predicted risk probabilities.

The analysis of logistic regression results and variance inflation factor values provides critical insights into the development of prehospital predictive models for cardiogenic shock. Logistic regression findings emphasize that Killip classification at presentation (Coef = 1.1394, *p*-value = 0.0000) is a highly significant predictor, reinforcing its pivotal role in early risk assessment. Additionally, ECG rhythm at presentation (Coef = 1.0267, *p*-value = 0.0188) emerges as another key variable, suggesting that specific electrocardiographic abnormalities may serve as early indicators of hemodynamic instability. Although other variables, including pain onset (Coef = 0.3753, *p*-value = 0.1185), age (Coef = 0.0188, *p*-value = 0.3489), and sex (Coef = 0.3764, *p*-value = 0.4649), do not reach statistical significance, their inclusion remains valuable for comprehensive patient stratification and refining risk estimation, as demonstrated in Table 3.

The VIF analysis, as shown in Table 4, confirms that none of the predictors exhibit multicollinearity, ensuring that each parameter contributes unique and non-redundant information to the model. Notably, Killip classification (VIF = 1.1415) and ECG rhythm at presentation (VIF = 1.1443) demonstrate low VIF values, reinforcing their stability and importance in prehospital risk assessment. This supports the integration of these variables into predictive modeling to enhance the early identification of high-risk patients before hospital admission.

The model utilizing all seven features demonstrated strong performance, achieving an accuracy of 77.42%, a sensitivity of 77.78%, and an AUC of 0.7735, which indicates high discriminative power. In contrast, the model restricted to the top three features exhibited a significant drop in performance, with an accuracy of 64.52%, sensitivity of 56.25%, and an AUC of 0.6517. These results suggest that the reduced feature set was notably less effective in distinguishing between high- and low-risk patients.

The confidence intervals further corroborate these findings, with the 95% CI for accuracy in the full-feature model ranging from 61.29% to 90.32% compared to 58.06% to 87.10% for the reduced model. The narrower and higher CI for the full-feature model reflects its superior stability and precision. Similarly, the F1-score’s confidence interval was wider and lower for the three-feature model, further supporting the conclusion that a larger, more diverse set of parameters enhances both predictive accuracy and consistency.

Statistical analysis indicates that reducing the number of features led to a decline in model performance. While the simplified model with only three features may be more interpretable, it compromises essential metrics like sensitivity and specificity, leading to a higher risk of misclassification. Conversely, the more comprehensive seven-feature model, selected using Random Forest feature importance and validated with variance inflation factor analysis, showed superior predictive accuracy and enhanced model stability.

Calibration evaluated through the Brier score provides additional confirmation of these findings. The seven-feature model exhibited a lower Brier score, indicating that its predicted probabilities corresponded more closely to actual outcomes compared to models with fewer features. This underscores the model’s reliability in estimating patient risk levels, which is essential for informed clinical decision-making. In contrast, poorer calibration observed in reduced-feature models may result in less accurate risk estimates, potentially affecting treatment strategies and patient outcomes.

In clinical practice, these findings underline the importance of incorporating a wider range of parameters for more effective patient risk assessment, particularly in prehospital and emergency settings where the early identification of high-risk patients is vital for timely intervention. A model with only a few features may fail to fully capture the complexities of cardiogenic shock, potentially resulting in less optimal triage decisions and poorer patient outcomes. The seven-feature model, incorporating a broader spectrum of patient characteristics, provides a more robust and comprehensive framework for early clinical decision-making.

Ultimately, this analysis highlights the critical balance between model simplicity and predictive power. While ease of interpretation is crucial, particularly in fast-paced clinical environments, the results from this study emphasize the importance of ensuring that predictive models not only remain accessible but also deliver precise, robust, and well-calibrated outputs for real-world medical applications.

#### 3.2.2. Emergency Department Evaluation Phase

Upon patient arrival at the emergency department, an additional set of key parameters is incorporated to refine the prediction of cardiogenic shock. This second phase of the model integrates 17 parameters from the prehospital phase, along with new biochemical and clinical markers, as shown in the methods described. The inclusion of biochemical markers into early risk assessment models for cardiogenic shock in the ED enhance predictive accuracy when combined with clinical and hemodynamic parameters. By analyzing their individual and combined contributions, the research could establish a biomarker-based scoring system, optimizing early diagnosis, treatment prioritization, and patient outcomes in acute cardiac care.

The set of key characteristics—Killip presentation, potassium (K), creatinine, age, CKI, EKG rhythm at presentation, and ALT—was identified through feature selection using RF analysis. Given the previous context, we utilized Random Forest as the primary predictive model for cardiogenic shock risk, while logistic regression was incorporated to determine the explanatory power of the selected features.

The Random Forest model demonstrated superior predictive performance compared to logistic regression, achieving a higher overall accuracy (74.19% vs. 64.52%, with 95% confidence intervals of [58.79%, 89.60%] for RF and [47.67%, 81.36%] for LR), indicating greater reliability in classification. RF also exhibited markedly higher sensitivity (94.44% vs. 77.78%), underscoring its stronger ability to correctly identify high-risk cardiogenic shock patients and minimize false negatives, a critical factor in acute care settings. While both models showed identical specificity (46.15%), highlighting limitations in correctly classifying low-risk patients, RF outperformed LR in the F1-score (0.6201 vs. 0.5793), reflecting a better balance between precision and recall. The area under the curve (AUC) was also higher for RF (0.8291 vs. 0.6752), suggesting better overall discrimination between high- and low-risk patients, as demonstrated in Table 5. Additionally, calibration analysis using the Brier score indicated better probability estimation for Random Forest (0.1737) compared to logistic regression (0.2164), further supporting the superior predictive reliability of the Random Forest model.

The McNemar test *p*-value of 0.3711 indicates no statistically significant difference in performance between the Random Forest and logistic regression models. This suggests that while the Random Forest model outperforms logistic regression in terms of accuracy, sensitivity, and F1-score, the difference is not large enough to be deemed statistically significant, meaning that both models exhibit similar overall performance when evaluated on this dataset. This finding reinforces the idea that both models may be used interchangeably in clinical settings, with the choice depending on the need for interpretability (LR) versus predictive power (RF).

From a statistical point of view, the table of logistic regression coefficients emphasizes the significance of certain clinical parameters in predicting cardiogenic shock. Killip presentation and EKG rhythm at presentation are identified as the most influential variables, with strong coefficients and low *p*-values (0.0000 and 0.0173, respectively), signifying their crucial role in outcome prediction. Potassium (K) and CKI also show some statistical significance, though their contributions are less robust, with potassium having a *p*-value of 0.3066 and CKI having a *p*-value of 0.0051, as shown in Table 6. In contrast, variables like creatinine, age, and ALT have high *p*-values, indicating they have minimal impact on the prediction of cardiogenic shock in the prehospital setting. This statistical analysis underscores the importance of certain clinical features in risk stratification while highlighting the focus of the model on key biomarkers and clinical indicators.

The variance inflation factor values in this table provide important insights into the level of multicollinearity among the predictor variables. A VIF value greater than one indicates that the variable is correlated with other variables in the model, which can potentially affect the stability of the regression coefficients. However, all VIF values in this table are below the commonly accepted threshold of two, suggesting minimal multicollinearity among the selected features. Specifically, creatinine has the highest VIF (1.6557), as demonstrated in Table 7, but it remains well below the cutoff point for concern. This indicates that the predictors used in the logistic regression model are not excessively correlated with one another, ensuring that the model’s estimates are stable and reliable. Thus, the predictors used in this model can independently contribute to the prediction of cardiogenic shock without significant redundancy.

In conclusion, the key characteristics identified through feature selection via Random Forest are critical for accurately evaluating the risk of cardiogenic shock in emergency settings. These selected parameters offer a comprehensive understanding of the patient’s condition, and their integration into predictive models improves the identification of high-risk patients, thereby supporting timely interventions and enhancing clinical outcomes in an emergency department.

#### 3.2.3. Cardiology Consultation Phase in Emergency Department

In the cardiology consultation phase within the emergency department, the inclusion of additional clinical and echocardiographic parameters plays a significant role in improving the prediction of outcomes for patients with acute cardiogenic shock or acute myocardial infarction. The integration of echocardiographic parameters, namely the LVEF at presentation, mitral regurgitation, right ventricular dysfunction, and pericardial effusion, provides valuable insight into the patient’s cardiac condition, enhancing the understanding of disease severity and prognosis.

As shown in Table 8, the LVEF at presentation, while clinically relevant, demonstrated only moderate predictive power for early cardiogenic shock in STEMI and was clearly outperformed by other clinical features. Focusing only on the LVEF may therefore lead to underestimating the patient’s risk. Instead, a more accurate and clinically meaningful risk assessment arises from combining multiple high-impact predictors.

In this context, the key parameters identified as most predictive during the cardiology consultation phase—Killip class at presentation, age, creatinine, CKI, potassium, AST, and EKG rhythm—were selected through Random Forest feature importance analysis. These variables, which reflect the patient’s overall physiological and cardiovascular condition, formed the basis of our Random Forest prediction model.

Compared to logistic regression, the Random Forest model demonstrated consistently stronger performance, highlighting the added value of integrating a broader set of clinical features beyond echocardiographic measures like the LVEF in predicting cardiogenic shock.

In the cardiology consultation phase analysis within the emergency department, both models performed well (Table 9) but Random Forest showed superior results across most evaluation metrics. Specifically, Random Forest achieved a higher accuracy (77.42%; 95% CI: [62.70%, 92.14%]) compared to logistic regression (74.19%; 95% CI: [58.79%, 89.60%]), suggesting better overall classification reliability.

The Random Forest model also demonstrated slightly higher sensitivity (75.00% vs. 70.00%) and matched specificity (81.82% for both models), indicating a marginally improved ability to identify high-risk patients without increasing false positives. Its F1-score (0.7826 vs. 0.7545) further supports its superior balance between precision and recall—important in high-risk clinical settings. The AUC value was also higher for Random Forest (0.9091) versus logistic regression (0.8273), demonstrating stronger discriminatory power. Furthermore, calibration assessed by the Brier score showed more favorable results for Random Forest (0.1498) than for logistic regression (0.1771), indicating a better alignment between predicted probabilities and actual outcomes. The McNemar test yielded a *p*-value of 1.0000, suggesting no statistically significant difference in classification performance between the two models. Nonetheless, the overall pattern of results supports Random Forest as a more robust and clinically reliable tool for risk stratification during cardiology consultations in an emergency setting.

Furthermore, the subsequent analysis will focus on the logistic regression coefficients and their statistical significance, offering insight into the logical contribution of each parameter to the prediction model and its relevance in assessing the risk of cardiogenic shock.

In Table 10, the Killip presentation coefficient indicates a strong, statistically significant contribution to the model, with a 95% confidence interval (CI) of [0.6150, 1.6376], emphasizing its pivotal role in predicting outcomes. Similarly, EKG rhythm at presentation (coefficient = 1.2979, *p*-value = 0.0096, CI = [0.3052, 2.2906]) also exhibits statistical significance, suggesting that variations in the patient’s initial rhythm at presentation are strongly associated with the likelihood of developing cardiogenic shock. Potassium (coefficient = 0.8889, *p*-value = 0.0208, CI = [0.1274, 1.6505]) is another significant parameter, showing its importance in risk stratification, while CKI (coefficient = −0.0004, *p*-value = 0.0596, CI = [−0.0008, 0.0000]) is close to significance, indicating its potential relevance but with a more borderline effect. In contrast, age (coefficient = 0.0277, *p*-value = 0.1448, CI = [−0.0099, 0.0652]) and creatinine (coefficient = −0.0575, *p*-value = 0.8723, CI = [−0.7658, 0.6508]) exhibit high *p*-values, suggesting minimal or no impact on the prediction of cardiogenic shock in this context. Finally, AST (coefficient = −0.0009, *p*-value = 0.4306, CI = [−0.0033, 0.0014]) also shows no significant contribution, further highlighting that some clinical markers may be less relevant in this specific risk assessment.

These statistical results underline the importance of certain clinical features, particularly Killip presentation and EKG rhythm, in predicting the likelihood of cardiogenic shock, while other parameters like age, creatinine, and AST provide little to no predictive value in this model.

However, to accurately predict the risk of cardiogenic shock, it is essential to consider all the identified parameters, as each one plays a distinct role in the overall model. For the model to be both robust and comprehensive, it is important to include all these factors, ensuring that no critical information is overlooked.

Next, the variance inflation factor for the selected variables will be analyzed to assess multicollinearity and ensure model stability.

The VIF values for each variable—Killip presentation, age, creatinine, CKI, potassium, AST, and EKG rhythm at presentation—are all below the threshold of concern (typically VIF > 5 or 10). These values, as demonstrated in Table 11, indicate minimal multicollinearity, suggesting that the selected features can contribute independently to the prediction model without introducing redundancy or instability. Therefore, the model’s predictions remain reliable and interpretable as we move forward with further evaluations.

In comparison to the emergency department evaluation phase, the predictive power of the models remains robust, with the primary difference being the inclusion of AST instead of ALT as a predictor. The models still share six common significant predictors, further confirming the validity and stability of the chosen predictors in both the emergency department phase and cardiology consultation phase.

Although the Random Forest model demonstrates solid predictive performance, further improvements in accuracy can be achieved by exploring additional machine learning algorithms.

This study applies and compares 11 ML classifiers to real-world data from patients with ST-segment elevation myocardial infarction complicated by cardiogenic shock (STEMI-CS), aiming to identify effective models for prognostic evaluation across critical phases of care.

The models were assessed using several key performance metrics such as accuracy, precision, recall, F1-score, and Matthews correlation coefficient, along with confusion matrices (true negatives—TNs; false positives—FPs; false negatives—FNs; and true positives—TPs). The predictive task was formulated as a binary classification problem, targeting the presence of cardiogenic shock, where Class 1 includes STEMI patients with CS present at admission and Class 0 refers to STEMI patients with CS post-admission.

In Table 12 is the performance evaluation of the 11 MLs on the PreHospital Care dataset (BD_ES_Prehospital.csv). Among all evaluated models, the ET classifier demonstrated the highest overall performance. It achieved an ACC of 0.9062, precision of 0.9078, recall of 0.9062, F1-score of 0.9061, and MCC of 0.8140, indicating both high predictive power and strong generalization. The NB Classifier also performed well, with an ACC of 0.8437, precision of 0.8450, recall of 0.8437, F1-score of 0.8435, and MCC of 0.6888. In contrast, the DT model showed the weakest performance, achieving the lowest accuracy of 0.625, likely due to overfitting and limited generalization capacity.

Table 13 presents the comparative performance metrics of 11 MLs applied to the Emergency Department dataset (BD_ES_ED.csv). Among all models, the SVM demonstrates the highest overall performance with an ACC of 78.12%, precision of 81.16%, recall of 78.12%, F1-score of 77.57%, and MCC of 59.21%. The NB and QDA classifiers show relatively strong and consistent results, each achieving 75% accuracy and comparable precision and recall values. However, the KNN classifier obtains the lowest performance with an ACC, precision, recall, and F1-score of 62.5% and an MCC of 25%.

Table 14 displays the performance metrics of 11 MLs applied to BD_ES_ED_CARDIOLOGIST.csv. RF shows the best performance with an ACC of 81.25% and consistent values across other metrics (precision: 83.33%; recall: 81.25%; F1-score: 80.95%; MCC: 64.54%). The lowest performance is observed again for KNN with an ACC, precision, recall, and F1-score of 62.5% and an MCC of 25%.

In the performance evaluation tables, the best-performing model for each metric is highlighted in green, the second-best in yellow, and the third-best in gray.

## 4. Discussion

An innovative approach is the development of stage-specific predictive models for cardiogenic shock risk evaluation in patients with STEMI, attempting to investigate how medical tests performed at different phases of the patient’s consultation could contribute to an earlier CS prediction. This approach provides a comprehensive understanding of disease progression across key clinical stages, from prehospital care to intensive care, and supports timely intervention strategies. In our study, a crucial step for building a reliable predictive model for CS risk is to identify the most relevant clinical and laboratory parameters that influence this risk at each stage. This would result in better risk stratification and personalized medical decision-making to mitigate this risk.

### 4.1. Key Prognostic Variables for Risk of Progression to Cardiogenic Shock in STEMI Patients

- Main Objective and Comparison with the Literature

Our study’s primary objective was to develop predictive models for the progression to CS in STEMI patients at various early stages of their management using machine learning (ML), specifically the Random Forest algorithm. In comparison with the existing literature, where most studies rely on classical statistical models like multivariate logistic regression, AI-based approaches are still limited, although they promise a better risk stratification and optimization of clinical decisions.

- Key Predictors of Progression to CS

Killip class proved to be the most powerful predictor of progression to CS, maintaining its relevance across all analyzed stages, from prehospital to ED and ED cardiology consultation. This highlights the importance of an early and integrated assessment of heart failure severity in STEMI patients. In addition to Killip class, other parameters such as age, time from pain onset, ECG rhythm, and biological markers like hemoglobin level, serum potassium, and transaminases were found to be important markers. These findings suggest not only a broader systemic involvement in the pathogenesis of cardiogenic shock, including renal, hepatic, and electrolyte imbalances, but also the impact of clinical factors on STEMI progression. Age influences cardiac reserve, time to presentation determines the extent of infarction, and ECG rhythm reflects ischemia severity and arrhythmic risk—all contributing to progression toward cardiogenic shock.

- Comparison with Existing Studies

Comparing our results with the existing literature, most studies on CS predictors rely on multivariate logistic regression, while ML-based studies are still rare and insufficiently validated. However, some recent research has explored ML models like XGBoost and LASSO regression for an early identification of STEMI and validated risk models on large patient datasets, such as The Shock Index Creatinine (SIC), supporting the importance of integrating renal biomarkers in predicting the progression to CS.

- Importance of Predictive Models in Prehospital Setting

A key finding of our study is the importance of predictive models in the prehospital phases, playing a critical role in early identification of STEMI patients at risk of CS and optimizing therapeutic decisions. The prehospital model is essential for the rapid triage of patients and efficient allocation of resources to the catheterization lab, Additionally, predictive models for the emergency room play an important role in risk stratification for CS; however, the emergency physician can independently assess the patient without the immediate presence of a cardiologist. This emphasizes the need for a standardized protocol in the emergency department that allows for rapid and accurate triage, optimizing the decision-making flow and reducing the time to initiate appropriate treatment.

- Phase-Specific Model Performance Insights

Different ML algorithms were selected according to the clinical phase to optimize predictive performance based on data availability and complexity. In the prehospital phase, the ET algorithm achieved the highest accuracy (90.62%) due to its robustness in handling heterogeneous clinical data—such as ECG findings, Killip class, and symptom onset—and its resilience to noise and missing values, which are common in early care settings. In the emergency department, the SVM delivered the best performance, offering clear decision boundaries between low- and high-risk patients once structured biochemical parameters (e.g., CKI, potassium, ALT) became available. The SVM was also efficient in classifying small, high-value datasets. During the cardiology consultation and interventional phases, Random Forest (RF) showed strong predictive capacity, with accuracies of 81.25% and 87.5%, respectively.

### 4.2. Necessity of Study on Predictive Models for Progression to Cardiogenic Shock in STEMI Patients

Most existing studies on CS prediction focus on identifying variables associated with mortality in patients who are already in this critical stage [46,47,48,49,50,51]. In contrast, our study extends the applicability of predictive models to a population at risk of developing CS, namely patients with STEMI, the group most vulnerable to this complication. Unlike previous approaches that analyze factors correlated with mortality, we focused on variables that predict progression toward CS, thereby enabling earlier, more targeted interventions.

The present study highlights the importance of selecting stage-specific machine learning models tailored to each clinical phase of STEMI patient management complicated by cardiogenic shock. Our results showed notable variations in model performance across different clinical stages. In the prehospital phase, the Extra Trees algorithm demonstrated superior performance due to its robustness in handling heterogeneous and noisy clinical data (e.g., ECG, Killip classification, pain onset). Upon patient arrival at the emergency department, the support vector machine (SVM) algorithm proved most effective by clearly distinguishing high-risk from low-risk patients, particularly when biochemical parameters (CKI, potassium, ALT) became numerically better defined. Moreover, clinical parameters identified as predictive by these models—such as Killip classification and biochemical markers (creatinine, CKI, potassium, hemoglobin)—have significant implications for early risk stratification, therapeutic decision-making, and personalized patient management.

CS presents with a wide range of clinical manifestations, which can delay diagnosis and, consequently, the early initiation of treatment. Additionally, the decision-making process is further complicated by the absence of specific biomarkers for CS. This underscores the need for personalized approaches and the involvement of multidisciplinary teams [52,53,54].

In the study conducted by El-Mughayyar D. et al., published in February 2025 in *CJC Open*, the authors evaluated the implementation of a multidisciplinary team for cardiogenic shock management (CS-Team), focusing on three key stages, namely early screening, team activation, and the use of invasive monitoring to guide therapy. Their results showed that early screening was documented in 74% of patients admitted to the coronary intensive care unit, with 6.9% ultimately diagnosed with cardiogenic shock. However, the study also reported a 26% failure rate in early screening, underscoring the need for even earlier and more refined risk stratification to improve the timely identification and management of patients at risk of cardiogenic shock [54]. Building upon the study by El-Mughayyar and colleagues, our research aims to further enhance risk stratification in order to minimize these screening failures as much as possible. To achieve this, we divided the patient’s clinical journey into three essential stages—prehospital care, emergency department assessment, and ED cardiology consultation—each designed to allow for dynamic reassessment and continuous risk evaluation according to the patient’s evolving clinical condition. This structured approach ensures that risk assessment is not static but rather adapts to the patient’s trajectory, thereby improving the early detection and management of cardiogenic shock.

### 4.3. Comparative Analysis of Predictive Models for Cardiogenic Shock Progression in STEMI Patients and Our Study

Since the introduction of the SCAI SHOCK staging classification, multiple validation studies have demonstrated a significant increase in mortality in the advanced stages of CS. However, most research has focused exclusively on patients who are already experiencing CS without considering the early stages of hemodynamic deterioration. This lack of data highlights the need for predictive models that are not limited to patients already diagnosed with CS but are applicable from the earliest phases of STEMI management, offering a dynamic and personalized approach for identifying high-risk patients. Moreover, to the best of the authors’ current knowledge, no published research to date has addressed the early detection of CS in a stepwise manner, analyzing each phase in the progression of a STEMI patient and interpreting all stages as part of a continuous clinical trajectory. Currently, the available data on the pre-shock stage as defined by the SCAI SHOCK classification are limited, and the use of machine learning to develop predictive models capable of anticipating this complication could mark the beginning of a new era in the field.

Therefore, our study proposes an innovative approach using ML models to dynamically analyze each stage of progression from STEMI to CS, thereby providing a predictive method with broad clinical applicability.

The most important predictive models are those in the prehospital phase and the emergency department considering that the prognosis of STEMI and the risk of complications, including CS, critically depend on the time required for reperfusion of the infarct-related coronary artery [55,56]. These models allow for the prioritization of patients for the catheterization lab, selecting those who are in critical condition or at high risk for unpredictable progression to CS, thereby facilitating rapid interventions and an optimal allocation of medical resources.

#### 4.3.1. Prehospital Care

To our knowledge, this is one of the first studies to develop a machine learning-based risk model specifically designed for prehospital use in STEMI patients, focusing on the earliest phase of care.

The prehospital care phase plays a crucial role in the early management of cardiogenic shock. The early identification of high-risk patients can significantly impact outcomes, especially in prehospital settings.

To refine our model, we assessed the impact of feature selection on predictive accuracy by comparing models using three versus seven parameters. In addition to evaluating model performance across different algorithms, this analysis addressed the practical trade-off between simplicity and comprehensiveness, which is especially relevant in time-sensitive emergency settings. This comparison aimed to determine whether a more simplified model, relying on a reduced set of parameters, could maintain sufficient discriminatory power or if a more comprehensive approach with additional features was necessary for optimal prediction. By examining this trade-off between model complexity and performance, we sought to strike a balance between clinical applicability and predictive reliability, ensuring that the model remains both efficient and effective in real-world settings.

The comparable performance of both models suggests that while Random Forest effectively captures non-linear interactions between variables, logistic regression remains a robust and reliable approach due to its interpretability and predictive power. This is supported by a large-scale study on 24,461 patients with acute decompensated heart failure, which demonstrated the utility of logistic regression in identifying individuals at high risk of cardiogenic shock in early clinical settings [57,58]. Building on this, integrating both models and further refining the feature selection process could enhance prehospital risk assessment, striking a balance between model complexity and usability. The relevance of such early intervention is emphasized by findings from another study that identified prehospital predictors of mortality in non-traumatic shock, including age, out-of-hospital cardiac arrest, and endotracheal intubation—all factors that could be incorporated into real-time decision support tools [59].A separate study validated a machine learning model that relied solely on vital signs for early shock detection, suggesting that simplified models can still deliver clinically meaningful predictions in prehospital environments [60]. Moreover, the similar performance of the two models, further confirmed by the McNemar test results, provides flexibility in selecting the most appropriate model for different clinical contexts. Importantly, this prehospital predictive approach may have the greatest impact, as it allows for early risk stratification and intervention before hospital arrival. 

A robust prehospital predictive model offers significant clinical advantages, particularly by enabling the early identification and prioritization of high-risk patients for expedited transfer to specialized cardiac centers equipped with advanced circulatory support systems such as extracorporeal membrane oxygenation (ECMO) and intra-aortic balloon pumps (IABPs). This approach is supported by a recent meta-analysis conducted by Max M. Meertens et al., which demonstrated that the combined use of veno-arterial ECMO (VA-ECMO) and IABPs in the management of cardiogenic shock following ST-elevation myocardial infarction (STEMI) was associated with significantly lower 30-day mortality compared to VA-ECMO alone [60]. By integrating such predictive tools into early care workflows, emergency medical services can shift from a reactive to a proactive approach, ensuring early hemodynamic stabilization and access to definitive, resource-intensive therapies for those most at risk.

Moreover, by integrating key predictors into early clinical workflows, prehospital teams can optimize early intervention strategies including tailored fluid resuscitation, vasopressor therapy, and anticoagulation, thereby ensuring hemodynamic stabilization prior to definitive reperfusion therapy. This approach is exemplified by the work of Allan Böhm and colleagues, who developed and validated the STOP SHOCK score—a prehospital risk assessment tool designed to identify patients at high risk for developing cardiogenic shock. Their study demonstrated that the early identification of high-risk patients based on clinical parameters enables frontline medical teams to implement proactive therapeutic measures aimed at maintaining hemodynamic stability before arrival at the hospital [19].

The early activation of interventional cardiology teams during the prehospital phase has demonstrated significant benefits in reducing treatment delays and improving outcomes in STEMI patients by ensuring timely reperfusion therapy, which is critical for improving survival in patients with evolving cardiogenic shock as shown by Savage et al. [61].

Given the life-threatening nature of cardiogenic shock, early risk stratification using key clinical predictors is essential for optimizing triage, prehospital management, and early therapeutic interventions. By leveraging machine learning models trained on clinically relevant variables, the prehospital phase can shift from a reactive to a proactive and targeted approach, ultimately reducing mortality and improving outcomes. In this context, the CShock model, proposed by Hu et al., serves as a strong example of how artificial intelligence can support the early identification of cardiogenic shock. Developed using deep learning techniques and validated on both the MIMIC-III database and an external NYU Langone Health cohort, CShock demonstrated superior predictive performance compared to traditional scoring systems, with AUROC values of 0.821 and 0.800, respectively. The model relies solely on routinely collected clinical data (vital signs, laboratory results), making it broadly applicable in various clinical environments and particularly valuable in supporting early, life-saving interventions.

Our goal was to identify a balance between clinical usability—especially in time-sensitive environments—and predictive reliability in real-world scenarios. This challenge was similarly addressed by Shen et al., who compared several machine learning models, as well as a nomogram designed to predict in-hospital mortality in patients with acute myocardial infarction complicated by cardiogenic shock. Their study demonstrated that models incorporating a larger number of relevant clinical predictors, including laboratory findings and comorbidities, achieved higher predictive accuracy (AUROC 0.869). These findings support the notion that while simplicity is important for bedside use, including a sufficient number of informative features enhances model reliability and real-world applicability [62].

Jaskiewicz F. and Zielinska M. are among the few authors who have analyzed this stage, investigating predictors of progression to CS in patients with ACS. Without distinguishing between NSTEMI and STEMI, they used univariate and multivariate analyses to identify clinical predictors specific to this life-threatening complication. Following their analysis, they developed a model based on multivariate regression, including two main parameters, namely pale skin and hyperglycemia > 11.1 mmol/L [63]. Our study presents significant improvements by using the Random Forest (RF) algorithm to build a model with higher predictive power, relying on commonly available parameters that can be easily accessed by any physician working in the prehospital setting and having first contact with the patient. Killip class, age, ECG rhythm, time from pain onset, sex, heart rate, and ST elevation in aVR on an ECG provide an early risk assessment for progression to cardiogenic shock in STEMI patients. However, validating our model on external datasets is essential to increase its generalizability. In the future, the relevant parameters discussed in our study could serve as a foundation for further analysis in NSTEMI patients, with the ultimate goal of comparing the two models.

Killip class was the strongest predictor in our study, consistently maintaining its relevance across all stages analyzed. In the prehospital phase, before any potential PCI, our findings align with existing studies. For example, in December 2023, Hyun Lee K. et al. published a study on a cohort of 6649 patients, demonstrating that those with higher Killip classes had a greater incidence of cardiogenic shock and higher 30-day mortality [5].

Currently, there are no studies that specifically analyze Killip class in the prehospital setting for STEMI patients [64]. However, some research has employed machine learning techniques such as XGBoost and LASSO regression for the early identification of STEMI. Although these studies did not directly aim to predict cardiogenic shock, their ability to detect STEMI in the prehospital phase is highly valuable, as rapid intervention can also help prevent progression to CS. Therefore, our study makes a significant contribution to both the management of STEMI and the prevention of cardiogenic shock. Taken together, these findings highlight the critical importance of prehospital risk stratification. By integrating clinically validated parameters into real-time predictive tools, frontline providers can initiate timely life-saving interventions even before hospital arrival, improving outcomes in high-risk STEMI patients.

Regarding the use of machine learning for predicting CS in the prehospital phase, we identified a single study conducted by Yang C. et al., published in 2023 in *Circulation*. In a cohort of 2736 patients, they analyzed prehospital predictors of CS using logistic regression (LR). Their findings support our conclusions, showing that heart rate and age can be easily used in the prehospital setting to predict CS, especially when combined with other factors. Yang C.’s model also included dialysis, diabetes mellitus, heart failure, blood pressure, prehospital cardiac arrest, and infarct type [65]. In our study, the Random Forest (RF)-based model enhanced CS prediction by incorporating Killip class, ECG rhythm, time from pain onset, and sex, alongside age and heart rate. As such, we recommend the combined use of LR and RF models to create robust STEMI-CS predictive tools.

A major advantage of our model is its potential use in the prehospital phase by emergency physicians from the very first medical contact—whether at the patient’s home or the location of the emergency call. By relying solely on immediately available parameters and not requiring the patient’s medical history, this model allows for rapid risk stratification and more efficient therapeutic decision-making.

#### 4.3.2. Emergency Department

In assessing STEMI patients in the emergency department, in addition to prehospital clinical variables (physical examination and ECG changes), we also included biomarkers, given their increasingly important role in the diagnosis and prognosis of CS [66]. Beyond the well-established biomarkers—such as pH, lactate, glucose, creatinine, troponin, procalcitonin, and proBNP—new biomarkers have been identified as potentially valuable for CS risk stratification. These include Ang-2, Copeptin, cDPP3, GDF-15, ADM, proANP, sST2, suPAR, Cystatin C, KIM-1, P-NGAL, IL-6, FGF-23, and P-PENK. Some of these biomarkers have been integrated into prognostic scoring systems, such as the CLIP score (Cystatin C, lactate, IL-6, NT-proBNP) and the CS4P score (L-FABP, B2MG, ALDOB, IC1) [18,67,68].

However, these biomarkers are difficult to collect and use in emergency settings, which limits their applicability in the emergency department for the risk stratification of progression to CS.

In our study, the RF model developed for the emergency department included the following parameters: Killip class, serum potassium, creatinine, age, CK, ECG rhythm, and ALT. While most ML-based STEMI-CS prediction studies focus on in-hospital phases, our study emphasizes the need to assess pre-shock risk at the point of emergency presentation, when intervention can still alter clinical trajectory, especially when the predictive model relies on common and easily accessible parameters.

Jantti T. et al. published a study on 178 patients, evaluating the significance of liver enzymes in CS, and concluded that an increase in ALT of more than 20% within the first 24 h is associated with higher 3-month mortality, independent of other parameters [69]. Moreover, Li J. et al., in a study conducted on 712 patients, demonstrated that elevated ALT levels are associated with higher in-hospital mortality in STEMI patients [70]. Our study supports the data in the literature, with ALT emerging as an early predictive marker for progression to cardiogenic shock in our patients, although its predictive power was lower than that of other parameters.

Regarding creatinine, Shariefuddin W. et al. published a study in 2024 involving 1349 patients with AMI, in which they validated the Shock Index Creatinine (SIC) as a reliable predictor of in-hospital mortality in AMI patients, including those with STEMI [71]. Ran P. et al. also updated this model specifically for STEMI patients, validating the SIC score in a cohort of 1907 post-PCI patients for both in-hospital and one-year mortality [72]. In addition, Kanabar K. et al., in a 2024 study, confirmed that creatinine level is a strong independent predictor of in-hospital mortality in STEMI-CS patients (*n* = 426) [73]. Furthermore, SCAI SHOCK validation studies include both increases in creatinine and elevated transaminases as indicators of CS progression. These findings support the need for a predictive assessment of creatinine using machine learning in STEMI patients at risk of developing CS.

A recent study published by Gao H. et al. on 3 March 2025 analyzed 112,363 patients with ACS and developed a risk model to predict adverse in-hospital events, including cardiogenic shock. They examined multiple factors, including CK, and concluded that age, history of diabetes mellitus, renal dysfunction, heart rate, shock index, and cardiac arrest at admission are independent predictors of severe events. The model with the highest predictive performance was based on logistic regression (LR), with an AUC of 0.822 (95% CI: 0.822–0.822) [74]. CK did not demonstrate significant predictive power in Gao H.’s model, possibly due to the diagnostic heterogeneity of the study population, which included the full spectrum of ACS patients. In contrast, our study focused exclusively on STEMI, in which CK proved to be a useful predictor of cardiogenic shock. This suggests a potential future research direction which involves analyzing ACS subgroups separately and developing predictive models tailored to each category.

Zweck E. et al. published a study in which serum potassium levels were found to be correlated with short-term mortality in patients with CS [75]. The association between low potassium levels and malignant ventricular arrhythmias is well known. However, in STEMI, acute myocardial ischemia leads to activation of the Na-K ATPase pump, which results in a decrease in intracellular potassium and its accumulation in the extracellular space. This shift promotes the occurrence of re-entry-based tachyarrhythmia [76].

#### 4.3.3. Emergency Department Cardiology Consult

The representative model for the on-call cardiologist, developed using the Random Forest (RF) algorithm, closely mirrors the model created for the emergency department, sharing the same predictive parameters with only minor differences in their order of importance.

Killip class remains the strongest predictor, followed by parameters that demonstrated similar importance, namely age, serum creatinine, creatine kinase (CK), and serum potassium. Slightly further behind is AST, which replaces ALT, and ECG rhythm.

This similarity between models can be explained by the fact that both the emergency department physician and the on-call cardiologist assess the patient within a very short time window, meaning that the level of suspicion for progression to cardiogenic shock rises almost simultaneously. This highlights the crucial role of the emergency physician in the decision-making process and as a key member of the cardiogenic shock management team (CS-team), independent of the on-call cardiologist’s presence [77,78,79].

The results of our study support the need for a standardized protocol in the emergency department, in which every STEMI patient is rapidly evaluated to rule out the risk of progression to CS. This assessment should be based not only on well-known parameters (e.g., hypotension) [80,81] but also on the predictors identified in our study—Killip class, creatinine, potassium, CK, AST, ALT, age, and ECG rhythm. According to our findings, this risk stratification process can be carried out independent of the on-call cardiologist, who may already be involved in the assessment of another patient suspected of developing CS.

In this context, optimizing diagnostic time is essential, enabling the early identification of CS risk, which can significantly improve outcomes. Support for this concept is also found in the study by Kwak M., which showed a 40 min reduction in time to vessel reperfusion when the emergency department physician notified the on-call cardiologist of the presence of STEMI. Furthermore, this protocol increased the likelihood of patients receiving PCI within the first 90 min from 25% to 50%. However, these improvements did not significantly impact the incidence of CS [82]. By extrapolating these findings, it can be stated that the interpretation of our study results, namely that the emergency department physician can independently predict the risk of cardiogenic shock, is supported by the existing medical literature.

Furthermore, considering the lack of predictive power of more specific parameters—such as the left ventricular ejection fraction and right ventricular involvement, which are assessed exclusively by the on-call cardiologist—we believe that dedicated studies focusing on these variables are warranted. Although these parameters are clinically important, their association with the risk of CS may be independent, which could potentially reduce the need to include them in predictive models [83,84].

Another important observation is the minimal difference between the models developed for the emergency department and the on-call cardiologist, which lies in the replacement of ALT with AST following cardiology consultation. ALT is more specific for the liver. A higher AST/ALT ratio was associated with a higher MI severity, with a total occlusion of a coronary artery based on Djakpo D. et al.’s study [85]. The fact that it is raised later and persists longer may be the reason that AST replaced ALT in this phase in the predictive models.

A complementary explanation may be that in CS, hepatic injury caused by the hypoperfusion of vital organs leads to an increase in AST, making it a later-stage marker of progression toward CS [14]. Supporting this hypothesis, Bai Z. et al. published a study demonstrating the usefulness of AST as a predictive marker for progression to cardiogenic shock in STEMI patients. However, their study employed the LASSO model and focused primarily on the late-stage prediction of CS [83] in contrast to our Random Forest (RF)-based model, which demonstrated the value of AST as an early predictor—even at the ED stage.

Although the LVEF at presentation would theoretically be expected to predict shock risk in ischemic CS, which characterizes our study population, it did not emerge as a strong predictor in our analysis. This finding aligns with the existing literature, showing that the LVEF is not always correlated with CS risk, particularly in non-ischemic conditions such as valvular disease or cardiomyopathies [86,87,88]. Even after excluding such etiologies, several factors may explain the LVEF’s limited predictive value. First, other clinical variables may carry greater weight in the acute phase, as they more directly reflect a patient’s metabolic and hemodynamic status [88]. Second, the timing of LVEF assessment—performed at presentation—may not capture evolving cardiac deterioration, particularly in patients who develop CS during hospitalization [86,89]. This is supported by our cohort’s balanced distribution between patients presenting with shock and those developing it later, suggesting that initial assessments may not fully reflect disease progression.

In conclusion for this phase, although echocardiographic parameters did not demonstrate predictive value for the development of CS in STEMI patients in our study, the role of the on-call cardiologist goes beyond performing the echocardiogram, as their presence is essential for comprehensive evaluation and timely management. Moreover, interdisciplinary collaboration between the cardiologist, emergency physician, and other involved specialties is crucial for optimizing the clinical pathway and improving patient outcomes.

Our findings reinforce the utility of phase-specific ML models in optimizing early risk stratification. In settings with limited access to invasive monitoring or dedicated CS teams, such tools can bridge gaps in care and guide timely therapeutic decisions.

### 4.4. Clinical Implications and Directions for Digital Implementation

This phase-specific analysis supports the transition toward dynamic and personalized management of patients with STEMI-CS, emphasizing the need for early triage tools applicable across diverse care environments.

The predictive models developed in this study, based on routinely available clinical and paraclinical parameters, can provide real-time support in various emergency settings. For instance, in the prehospital phase, emergency medical teams could use these models to identify high-risk STEMI patients from the first point of contact and prioritize direct transfer to tertiary centers equipped with interventional cardiology and mechanical circulatory support. For example, if a patient presents with prolonged chest pain, Killip class ≥ II, and an abnormal ECG rhythm, a mobile application used by the ambulance crew or dispatch center could automatically compute a risk score and trigger a “high-risk STEMI” alert. This would guide the decision to bypass intermediate centers and transfer the patient directly to a specialized facility, minimizing delays to reperfusion. In this way, the model enables rapid, standardized, and data-driven triage even before hospital arrival.

In the emergency department, automatic alerts generated by the model scores could accelerate cardiology consultations, reduce door-to-balloon time, and enable early activation of the catheterization laboratory—especially in high-volume centers with limited resources.

These predictive models—based on accessible and easily collected data—can also be integrated into electronic health record (EHR) systems or implemented as clinical decision support tools (CDSS). For example, automated alerts could notify emergency physicians of a patient’s high risk of progression to cardiogenic shock, triggering early cardiology involvement or prioritization for invasive procedures. If, for instance, a STEMI patient presents with Killip class ≥ II, elevated serum creatinine, and hyperkalemia, the system could automatically recognize this risk profile and generate a “high-risk” alert. This would support the timely activation of the catheterization lab, urgent specialist consultation, or transfer to a center with advanced circulatory support. Simplified versions of these models could also be integrated into mobile applications or ambulance dispatch systems, enabling prehospital teams to perform real-time triage and initiate therapeutic interventions before hospital arrival. Such an integration would support real-time clinical decision-making and reduce delays in the management of high-risk patients.

In the future, we aim to translate these findings into a simplified clinical risk score that can be used independently or as part of a digital tool—either as a mobile application or integrated into hospital infrastructure. Such a tool would allow for rapid bedside risk estimation and the automatic generation of high-risk alerts, supporting physicians in anticipating cardiogenic shock and enabling early intervention in high-risk STEMI patients. However, successful integration into existing hospital systems may face challenges related to interoperability, workflow compatibility, and the need for clinician training and acceptance.

### 4.5. Lessons Learned

- Phase-specific predictive modeling enables a more granular understanding of cardiogenic shock risk in STEMI patients.
- Early identification of key clinical parameters—such as Killip class, creatinine, potassium, ECG rhythm, and symptom onset—can significantly improve triage decisions.
- Prehospital and emergency department models are critical for timely reperfusion and resource allocation, especially in settings with limited catheterization lab access.
- Random Forest and Extra Trees algorithms performed robustly in early phases, highlighting their potential for clinical integration.
- Predictive tools relying on simple, routine data can be feasibly implemented into standard workflows, including mobile applications and clinical alert systems.

## 5. Limitations

This study has several limitations that should be acknowledged, as follows:

- Sample Size and External Validation: As a retrospective, single-center study, these findings are also subject to potential selection and information biases. To enhance generalizability, future research should include larger, multicenter cohorts or validate the model on external datasets. Statistical findings should be interpreted accordingly and confirmed by more extensive prospective research. We acknowledge that the exclusion of NSTEMI and unstable angina patients may limit the generalizability of our findings. However, this methodological decision was made to enhance model robustness by focusing on STEMI patients, who follow a more standardized and time-sensitive emergency care pathway. Future studies will aim to expand this approach to other ACS subtypes with appropriate modeling strategies.
  Additionally, the current sample size and low number of cardiogenic shock events within subgroups limited the feasibility of conducting statistically robust subgroup analyses (e.g., by age, sex, infarct location, or comorbidity profile). Performing such analyses under these constraints could result in unstable estimates or misleading interpretations. This limitation will be addressed in future multicenter studies, where stratified performance evaluation across clinically relevant subgroups will be feasible.
- Lack of Ethnic Diversity: The study population consists exclusively of Caucasian patients, which may restrict the applicability of the model to other ethnic groups. Further validation in more diverse populations is necessary to broaden its clinical utility.
- Geographical Context: This study was conducted in an Eastern European country, where access to advanced mechanical circulatory support is more limited compared to Western Europe. To strengthen the predictive model’s robustness, validation on international datasets is required. Future research may also include subgroup analyses by geographic region or healthcare system characteristics to better understand potential disparities in model performance across different clinical environments.
- Socioeconomic Factors: The study was performed in a Romanian center of excellence, where patients have access to more resources than those treated in smaller regional hospitals. This may affect the model’s applicability in different healthcare settings. A subgroup analysis comparing STEMI-CS patients from high-resource centers with those from smaller hospitals could provide further insight into these disparities.
- Missing Data for Key Clinical Variables: Several clinically relevant parameters—such as NT-proBNP, lactate, blood pressure, oxygen saturation, and signs of hypoperfusion—were excluded from the final analysis due to incomplete data, particularly during early phases of care. Although these variables are known to contribute to cardiogenic shock risk prediction, including them would have introduced bias. Future prospective studies should incorporate systematic data collection to evaluate their predictive value more accurately.

Despite these limitations, our study presents a simple and applicable predictive model that can be further refined and expanded through future multicenter and international research.

## 6. Future Research Directions

The predictive models developed in this study still require external validation and could provide a valuable foundation for future multicenter investigations. Conducting such research across diverse patient populations and healthcare systems would help elucidate the impact of geographic and systemic disparities on the progression and management of STEMI-related cardiogenic shock.

This study represents an interdisciplinary collaboration between engineers and clinicians, combining medical expertise with artificial intelligence techniques to improve the assessment and early prediction of cardiogenic shock [29]. A crucial direction for future research lies in translating these AI-driven approaches into routine clinical practice by developing digital platforms that can process patient data and generate real-time risk predictions. In parallel, another important next step involves the derivation of a simplified clinical risk score based on the most predictive variables identified by the AI models. Such a score would support bedside decision-making in emergency settings and could serve as the foundation for mobile or EHR-integrated applications, enhancing practical adoption. Such tools have the potential to enhance early detection, reduce human error, and support more precise and timely medical decision-making. Moreover, a future integration of patient-reported outcome measures (PROMs) and patient experience measures (PREMs) may provide a more comprehensive understanding of the long-term impact of cardiogenic shock, aligning with the principles of personalized, patient-centered care [28,43,90]. This approach helps bridge the gap between clinical outcomes and patient perspectives and reinforces the growing emphasis on patient-centered medicine.

Additionally, expanding predictive models to include advanced imaging parameters (e.g., echocardiography, cardiac MRI) and specific biomarkers (e.g., NT-proBNP, high-sensitivity troponin) could further enhance diagnostic performance and risk stratification accuracy. Equally important is the need for prospective validation of these models in real-world clinical environments. Embedding them into clinical workflows and assessing their impact on physician behavior, treatment timelines, and patient outcomes will be essential for successful implementation.

**Figure 6:**
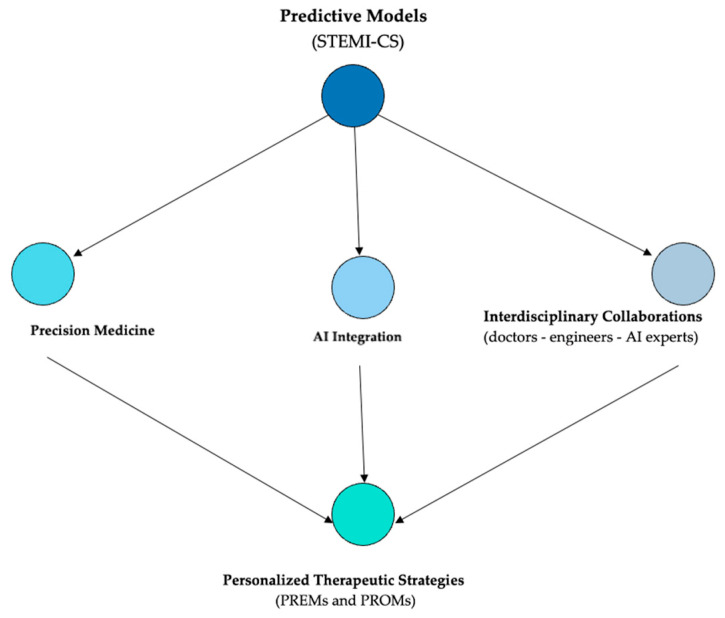
Future directions for treating STEMI-CS patients.

A schematic overview of the key concepts discussed in this section is illustrated in Figure 6, showing the conceptual framework of our study, centered around the development of predictive models for STEMI-CS patients. These models serve as the foundation for three essential and interconnected components, namely precision medicine, interdisciplinary collaboration, and AI integration. Artificial intelligence enables dynamic and adaptive risk assessment, while collaborations between clinicians, engineers, and AI specialists facilitate the clinical translation of predictive tools. Precision medicine is supported by individualized patient data, guiding the selection of the most appropriate therapeutic approach. Together, these elements converge to support personalized treatment strategies that incorporate both clinical indicators and patient-reported outcomes.

Despite the growing potential of these approaches, several critical gaps in the current knowledge warrant further investigation and represent essential directions for future research. These include the lack of extensive, multicenter, international databases, the absence of specific biomarkers for early CS detection, and the pressing need for tailored, patient-centered risk stratification algorithms [67,68,91,92,93,94]. In spite of these limitations, our findings may offer valuable insights for regions with limited access to mechanical circulatory support (MCS) and the restricted availability of catheterization laboratories, supporting improved STEMI-CS management in resource-constrained settings.

## 7. Conclusions

In conclusion, our study proposes a straightforward and practical predictive approach based on commonly available clinical and paraclinical parameters to assess the risk of progression to cardiogenic shock in STEMI patients. The predictive model can be applied from the first medical contact and extended across various stages of care, including the prehospital phase, emergency department, and cardiology consultation in the ED.

The most important clinical implication of this model is its ability to accurately triage truly high-risk patients and prioritize their transfer to the catheterization laboratory for prompt reperfusion therapy. This can be achieved using simple clinical parameters such as Killip class, age, ECG rhythm, and time from pain onset, as well as paraclinical markers like potassium, creatinine, and CKI. Given its simplicity and accessibility, this tool has the potential to support earlier intervention and improve outcomes in high-risk patients. However, this single-center study with a moderate sample size requires external validation in larger, multicenter cohorts to confirm the robustness and generalizability of the model across diverse clinical settings. As part of our ongoing research, we aim to extend dynamic risk modeling into post-reperfusion phases, particularly within interventional cardiology and intensive cardiac care settings, enabling continuous reassessment and real-time clinical decision support. In addition, we are considering the integration of this model into electronic health records, clinical alert platforms, or mobile applications to enhance its practical utility and support its implementation in routine clinical practice.

## Figures and Tables

**Table 1:** Descriptive statistics of the cohort.

STEMI—CS (158 Patients)			
STEMI patients with CS present on admission 87 patients (55.1%)		STEMI patients with CS post-admission 71 patients (44.9%)	
Females	Males	Females	Males
43 patients (49.43%)	44 patients (50.57%)	17 patients (24%)	51 patients (76%)
Cardiovascular risk factors		Cardiovascular risk factors	
Diabetes mellitus 23 (26.43%)		Diabetes mellitus 27 (38.02%)	
Hypertension 31 (36.78%)		Hypertension 55 (77.46%)	
Smoking 27 (31.03%)		Smoking 30 (49.29%)	
Dyslipidemia 65 (74.71%)		Dyslipidemia 50 (71.83%)	

**Table 2:** Model performance comparison of the prehospital phase. AUC: area under the curve.

Model	Accuracy	Sensitivity	Specificity	F1-Score	AUC	Brier Score
Random Forest	0.7742	0.7778	0.7692	0.7735	0.7735	0.1927
Logistic Regression	0.7419	0.7222	0.7692	0.7450	0.8248	0.1738

**Table 3:** Coefficients (logistic regression).

Parameter	Coefficient	*p*-Value	95% Confidence Interval
Killip at Presentation	1.1394	0.0000	[0.6708, 1.6080]
Age	0.0188	0.3489	[−0.0210, 0.0587]
ECG Rhythm at Presentation	1.0267	0.0188	[0.1614, 1.8920]
Pain Onset	0.3753	0.1185	[−0.1007, 0.8512]
Sex	0.3764	0.4649	[−0.6435, 1.3963]
HR at Presentation	−0.0009	0.9133	[−0.0165, 0.0148]
ST Elevation in aVR	−0.1368	0.8899	[−2.0937, 1.8201]

**Table 4:** Variance inflation factor (VIF).

Parameter	VIF
Killip at Presentation	1.1415
Age	1.2938
ECG Rhythm at Presentation	1.1443
Pain Onset	1.1968
Sex	1.2141
HR at Presentation	1.0706
ST Elevation in aVR	1.0768

**Table 5:** Model performance comparison of the emergency department evaluation phase.

Model	Accuracy	Sensitivity	Specificity	F1-Score	AUC	Brier Score
Random Forest	0.7419	0.9444	0.4615	0.6201	0.8291	0.1737
Logistic Regression	0.6452	0.7778	0.4615	0.5793	0.6752	0.2164

**Table 6:** Coefficients (logistic regression).

Parameter	Coefficient	*p*-Value	95% Confidence Interval
Killip Presentation	1.1891	0.0000	[0.6680, 1.7102]
K (Potassium)	0.3240	0.3066	[−0.3037, 0.9518]
Creatinine	−0.0429	0.9123	[−0.8136, 0.7279]
Age	0.0209	0.3201	[−0.0207, 0.0626]
CKI	−0.0006	0.0051	[−0.0010, −0.0002]
Electrocardiogram Rhythm at Presentation	1.6462	0.0173	[0.2770, 3.0155]
ALT (Alanine Aminotransferase)	−0.0001	0.9747	[−0.0042, 0.0041]

**Table 7:** Variance inflation factor (VIF).

Parameter	VIF
Killip Presentation	1.1229
K (Potassium)	1.4220
Creatinine	1.6557
Age	1.1044
CKI	1.0257
EKG Rhythm at Presentation	1.1188
ALT	1.2160

**Table 8:** Features by importance (Random Forest) of the cardiology consultation phase.

Feature	Importance Score	Rank (of 33 Variables)	Interpretation
Killip Presentation	0.9922	1	Most predictive; reflects clinical severity
Age	0.5915	2	Important demographic risk factor
Creatinine	0.5891	3	Indicator of renal function and systemic status
CKI	0.5784	4	Reflects severe myocardial damage and dysfunction
Potassium	0.5758	5	Electrolyte balance linked to arrhythmia risk
AST	0.4723	6	Marker of tissue injury
EKG Rhythm at Presentation	0.4409	7	Reflects electrical instability
LVEF at Presentation	0.2524	19	Moderate predictor; less informative alone

**Table 9:** Model performance comparison of the cardiology consultation phase.

Model	Accuracy	Sensitivity	Specificity	F1-Score	AUC	Brier Score
Random Forest	0.7742	0.7500	0.8182	0.7826	0.9091	0.1498
Logistic Regression	0.7419	0.7000	0.8182	0.7545	0.8273	0.1771

**Table 10:** Coefficients (logistic regression).

Parameter	Coefficient	*p*-Value	95% Confidence Interval
Killip Presentation	1.1263	0.0000	[0.6150, 1.6376]
Age	0.0277	0.1448	[−0.0099, 0.0652]
Creatinine	−0.0575	0.8723	[−0.7658, 0.6508]
CKI	−0.0004	0.0596	[−0.0008, 0.0000]
Potassium	0.8889	0.0208	[0.1274, 1.6505]
AST	−0.0009	0.4306	[−0.0033, 0.0014]
EKG Rhythm at Presentation	1.2979	0.0096	[0.3052, 2.2906]

**Table 11:** Variance inflation factor (VIF).

Parameter	VIF
Killip Presentation	1.1268
Age	1.0959
Creatinine	1.5216
CKI	1.1398
Potassium (K)	1.4051
AST	1.2527
EKG Rhythm at Presentation	1.1134

**Table 12:** The performance evaluation of the 11 MLs on the prehospital care phase.

MLs	ACC	Precision	Recall	F1-Score	MCC	TN	FP	FN	TP
ET	0.90625	0.907843	0.90625	0.906158	0.814092	14	2	1	15
RF	0.78125	0.791498	0.78125	0.779310	0.572656	11	5	2	14
DT	0.62500	0.633333	0.62500	0.619048	0.258199	8	8	4	12
QDA	0.75000	0.766667	0.75000	0.746032	0.516398	10	6	2	14
NB	0.84375	0.845098	0.84375	0.843597	0.688847	14	2	3	13
SVM	0.75000	0.750000	0.75000	0.750000	0.500000	12	4	4	12
LR	0.71875	0.719608	0.71875	0.718475	0.438357	11	5	4	12
RC	0.68750	0.690476	0.68750	0.686275	0.377964	10	6	4	12
GBC	0.75000	0.753968	0.75000	0.749020	0.503953	11	5	3	13
ADA	0.68750	0.690476	0.68750	0.686275	0.377964	10	6	4	12
KNN	0.68750	0.687500	0.68750	0.687500	0.375000	11	5	5	11

ET: Extra Trees Classifier; RF: Random Forest; DT: Decision Tree; QDA: Quadratic Discriminant Analysis; NB: Naive Bayes; SVM: Support Vector Machine; LR: Logistic Regression; RC: Ridge Classifier; GBC: Gradient Boosting Classifier; ADA: AdaBoost Classifier; KNN: K-Nearest Neighbors.

**Table 13:** The performance evaluation of the 11 MLs on the emergency department phase.

MLs	ACC	Precision	Recall	F1-Score	MCC	TN	FP	FN	TP
ET	0.62500	0.626984	0.62500	0.623529	0.251976	11	5	7	9
RF	0.75000	0.753968	0.75000	0.749020	0.503953	11	5	3	13
DT	0.62500	0.626984	0.62500	0.623529	0.251976	9	7	5	11
QDA	0.75000	0.790909	0.75000	0.740891	0.539360	9	7	1	15
NB	0.75000	0.790909	0.75000	0.740891	0.539360	15	1	7	9
SVM	0.78125	0.811688	0.78125	0.775776	0.592157	10	6	1	15
LR	0.71875	0.726721	0.71875	0.716256	0.445399	10	6	3	13
RC	0.75000	0.766667	0.75000	0.746032	0.516398	10	6	2	14
GBC	0.68750	0.690476	0.68750	0.686275	0.377964	10	6	4	12
ADA	0.75000	0.750000	0.75000	0.750000	0.500000	12	4	4	12
KNN	0.62500	0.625000	0.62500	0.625000	0.250000	10	6	6	10

**Table 14:** The performance evaluation of the 11 MLs on the dataset from the cardiology consultation phase in the emergency department.

MLs	ACC	Precision	Recall	F1-Score	MCC	TN	FP	FN	TP
ET	0.62500	0.633333	0.62500	0.619048	0.258199	8	8	4	12
RF	0.81250	0.833333	0.81250	0.809524	0.645497	11	5	1	15
DT	0.71875	0.726721	0.71875	0.716256	0.445399	10	6	3	13
QDA	0.75000	0.790909	0.75000	0.740891	0.539360	9	7	1	15
NB	0.75000	0.790909	0.75000	0.740891	0.539360	15	1	7	9
SVM	0.78125	0.811688	0.78125	0.775776	0.592157	10	6	1	15
LR	0.71875	0.726721	0.71875	0.716256	0.445399	10	6	3	13
RC	0.75000	0.766667	0.75000	0.746032	0.516398	10	6	2	14
GBC	0.68750	0.690476	0.68750	0.686275	0.377964	10	6	4	12
ADA	0.75000	0.750000	0.75000	0.750000	0.500000	12	4	4	12
KNN	0.62500	0.625000	0.62500	0.625000	0.250000	10	6	6	10

## Data Availability

The original data presented in the study are openly available on Figshare at https://doi.org/10.6084/m9.figshare.28787594.

## Abbreviations

The following abbreviations are used in this manuscript:

AI	Artificial intelligence
ACS	Acute coronary syndrome
ALT	Alanine aminotransferase
AST	Aspartate aminotransferase
AMI	Acute myocardial infarction
CI	Confidence intervals
CICU	Cardiac intensive care unit
CS	Cardiogenic shock
CS-Team	Team-based cardiogenic shock
CKD	Chronic kidney disease
DES	Drug eluting stent
DM	Diabetes mellitus
DT	Decision tree
ECG	Electrocardiographic
EHR	Electronic health record
ET	Extra Trees
GBC	Gradient boosting
HTN	Hypertension
KNN	K-nearest neighbors
LDH	Lactic acid dehydrogenase
LR	Logistic regression
LR	Logistic regression
MCC	Matthews correlation coefficient
MCS	Mechanical circulatory support
ML	Machine learning
NSTEMI	Non ST-elevation myocardial infarction
NB	Naïve Bayes
P-E-I-CI	Prioritization and evolving ischemia in cardiogenic instability
PREMs	Patient-reported experience measures
PROMs	Patient-reported outcome measures
QDA	Quadratic discriminant analysis
RF	Random Forest
SCAI SHOCK	Society for Cardiovascular Angiography and Interventions Shock
STEMI	ST-elevation myocardial infarction
SVM	Support vector machine
VIF	Variance inflation factor
WBC	White blood cell
